# Left Atrial Epithelioid Angiosarcoma Presenting As Recurrent Stroke: A Case Report

**DOI:** 10.7759/cureus.96455

**Published:** 2025-11-09

**Authors:** Yoshimi Tamura, Tadashi Kitamura, Masaomi Fukuzumi, Yusuke Motoji, Kagami Miyaji

**Affiliations:** 1 Cardiovascular Surgery, Kitasato University School of Medicine, Sagamihara, JPN; 2 Cardiovascular Surgery, Jichi Medical University, Shimotsuke, JPN; 3 Cardiovascular Surgery, National Center for Global Health and Medicine, Tokyo, JPN

**Keywords:** cardiac angiosarcoma, cardiac tumor, cerebral infarction, epithelioid angiosarcoma, minimally invasive cardiac surgery

## Abstract

A 74-year-old man presented with recurrent cerebellar infarction and progressive anemia. The workup revealed a small jejunal tumor and a mobile mass in the left atrium. Urgent minimally invasive cardiac surgery was performed, and histopathology confirmed epithelioid angiosarcoma. Subsequent small bowel resection revealed a similar tumor. Despite chemotherapy and radiotherapy, bone metastases progressed, and the patient succumbed to the disease eight months postoperatively. The cardiac tumor was smaller than typically reported, suggesting early detection prompted by embolic events. Nevertheless, the clinical course was aggressive, highlighting the high malignancy of epithelioid angiosarcoma and the limited effectiveness of current therapies. Accumulation of further clinical experience is essential to improve outcomes in this rare cardiac tumor.

## Introduction

Cardiac tumors are predominantly benign, with only approximately 20% classified as malignant. Most malignant cardiac tumors are metastatic, while primary cardiac tumors are extremely rare, with an estimated incidence of 0.001%-0.03% [1]. Among primary malignant cardiac tumors, sarcomas are the most common, with angiosarcoma representing the predominant subtype. Cardiac angiosarcomas typically arise in the right atrium and are often diagnosed due to symptoms such as cardiac tamponade or dyspnea. In contrast, left atrial angiosarcomas are rare, and presentations involving cerebral infarction are exceptionally uncommon. Epithelioid angiosarcoma, a rare subtype of angiosarcoma, is generally associated with an extremely poor prognosis, with median survival reported to be only several months in most cases. Even when detected early, management remains challenging due to its aggressive nature.

## Case presentation

**Figure 1:**
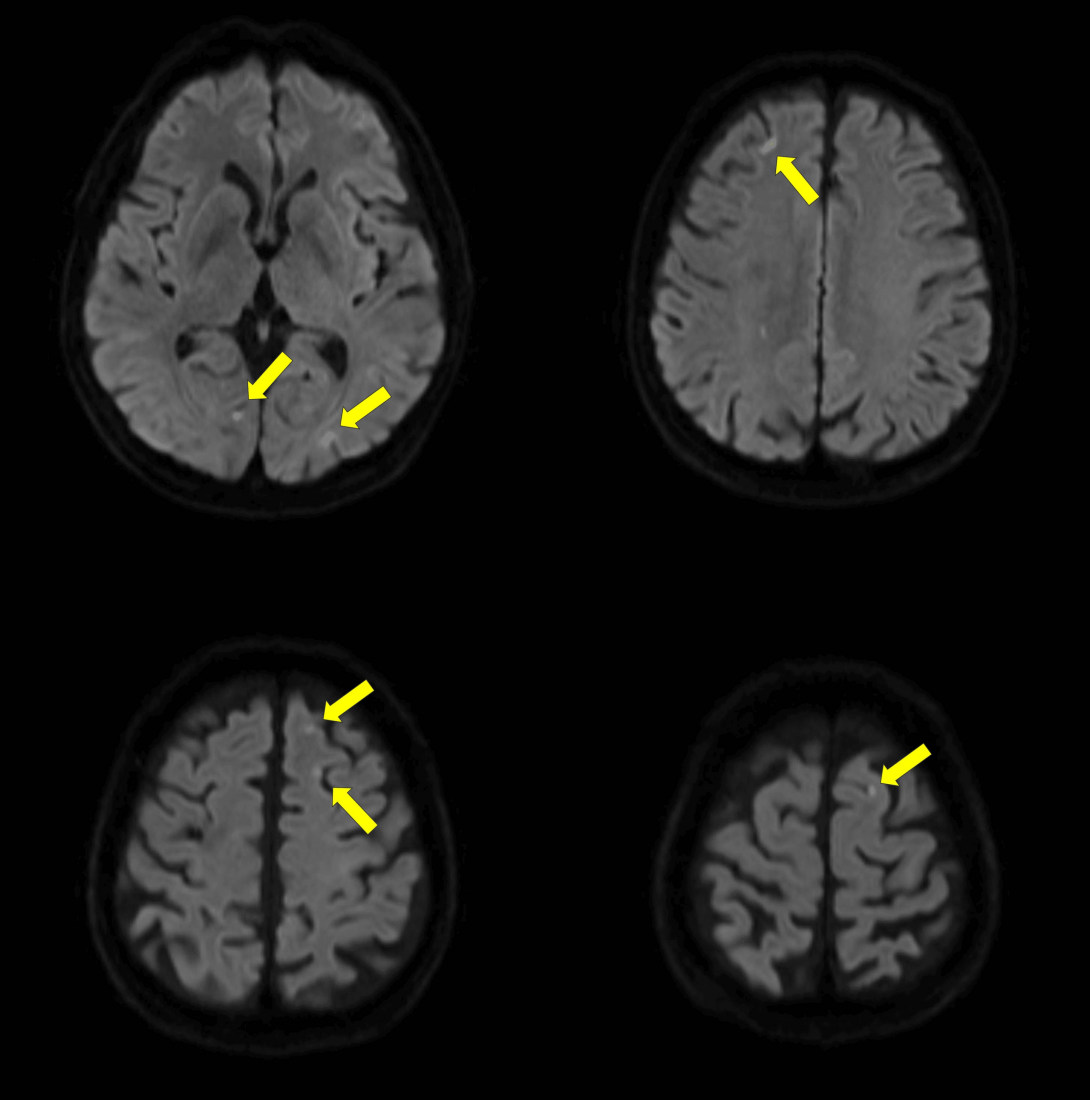
MRI showing bilateral cerebellar infarctions (arrows indicate the lesions).

A 74-year-old man presented with ataxia of the left upper limb at the referring hospital. Head magnetic resonance imaging (MRI) revealed bilateral cerebellar infarctions (Figure 1). His symptoms improved with rehabilitation, and he was discharged on clopidogrel (75 mg/day). Four months later, he developed severe dizziness and returned to the hospital, where he was diagnosed with recurrent cerebellar infarction. His hemoglobin level was 7.0 g/dL, and clopidogrel was discontinued. However, his anemia worsened, with hemoglobin levels dropping to 4.6 g/dL during hospitalization. A fecal occult blood test was positive, suggesting gastrointestinal bleeding as a potential cause of anemia. Although upper endoscopy and colonoscopy showed no apparent bleeding lesions, he was referred to our hospital for further evaluation. Table 1 shows the laboratory findings on initial presentation.

**Table 1:** Laboratory findings on initial presentation. CRP: C-reactive protein; AST: aspartate transaminase; ALT: alanine transaminase

Parameter	Value	Normal range
Hemoglobin	8.4 g/dL	13.7-16.8 g/dL
White blood cells	5,500/µL	3,300-8,600/µL
Platelets	21.8 × 10^4^/µL	15.8-34.8 × 10^4^/µL
Albumin	3.7 g/dL	4.1-5.1 g/dL
Creatinine	0.83 mg/dL	0.65-1.07 mg/dL
AST	27 U/L	8-38 U/L
ALT	28 U/L	4-44 U/L
CRP	0.24 mg/dL	0-0.30 mg/dL

**Figure 2:**
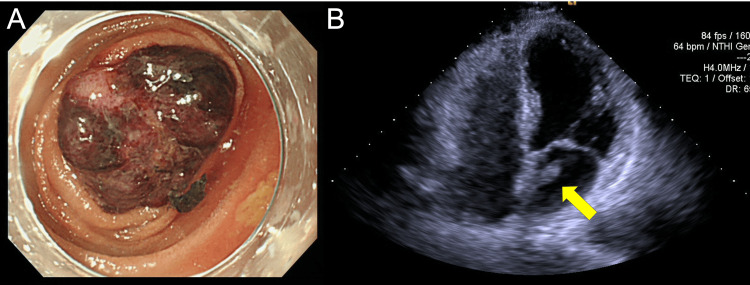
(A) Preoperative enteroscopy showing a 30-mm elevated lesion in the jejunum. (B) Preoperative transthoracic echocardiography revealing a mobile mass in the left atrium (arrow).

Enteroscopy revealed an irregularly elevated lesion measuring 30 mm in the middle jejunum (Figure 2A). Anemia was suspected to be secondary to bleeding from the jejunal tumor. A biopsy from this site was nondiagnostic, and poorly differentiated adenocarcinoma and sarcoma were considered in the differential diagnosis. Small bowel resection was planned; however, one month later, the patient developed left-sided hemiplegia and was admitted to our hospital with a diagnosis of a third cerebral infarction. Echocardiography, performed to investigate the embolic source, revealed a mobile mass measuring 15 × 10 mm in the left atrium, raising suspicion of a myxoma or hemangioma (Figure 2B and Video 1). Given the high likelihood that the tumor was the source of recurrent cerebral emboli, urgent surgical resection was deemed necessary.

**Video 1:** Preoperative transesophageal echocardiography showing a mobile mass in the left atrium.

**Figure 3:**
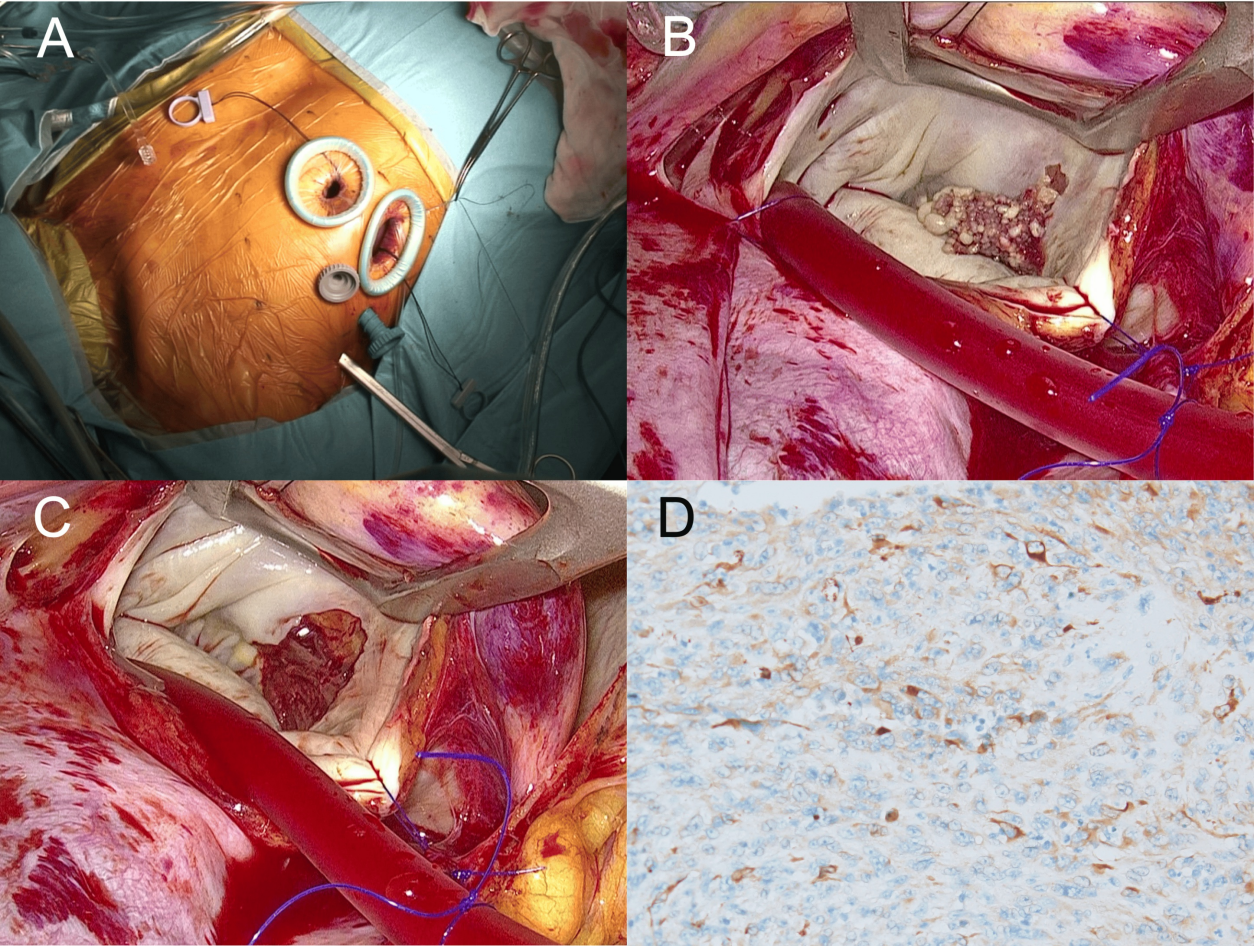
(A) Intraoperative image of minimally invasive tumor resection. (B, C) The tumor appeared miliary and was resected from within the myocardium. (D) Immunohistochemistry demonstrating positivity for AE1/AE3.

The patient underwent minimally invasive tumor resection six days after admission. Cardiopulmonary bypass (CPB) was established via the right internal jugular vein, right femoral vein, and femoral artery. The procedure was performed through four ports using a transthoracic aortic clamp (Figure 3A). The tumor appeared miliary and fragile, clearly distinct from a typical myxoma. It was excised with a 3 mm margin, with the resection extending into the myocardial layer of the left atrium. On the annular side, resection was performed at the mitral annulus. The atrial endocardial defect was left unrepaired with a patch due to concerns about inducing atrioventricular block (Figures 3B, 3C). The total operative time was 245 minutes, and the CPB time was 126 minutes (Video 2). No gastrointestinal bleeding or worsening of anemia occurred during hospitalization, and the patient was discharged 12 days postoperatively.

**Video 2:** Intraoperative footage of minimally invasive cardiac surgery.

Histopathological examination revealed positivity for endothelial markers CD31 and CD34, as well as the epithelial marker AE1/AE3, confirming the diagnosis of epithelioid angiosarcoma (Figure 3D). Fifty-three days after cardiac surgery, the patient underwent small bowel resection, with pathological findings consistent with epithelioid angiosarcoma, similar to the cardiac tumor. He was discharged six days postoperatively but returned one month later with complaints of lower back pain. MRI revealed bone metastases in the thoracic and lumbar spine. Chemotherapy with weekly paclitaxel (60 mg) and palliative radiotherapy were administered; however, during the third course of chemotherapy, new metastases developed in the upper thoracic spine, accompanied by a pathological fracture. Subsequently, systemic therapy was switched to pazopanib (400 mg/day), but treatment had to be discontinued after only two days because of intolerable adverse effects, including severe nausea. His general condition deteriorated, and he died of sepsis due to a urinary tract infection eight months and 12 days (256 days) after cardiac surgery. Throughout this period, echocardiography showed no evidence of local intracardiac recurrence.

## Discussion

Epithelioid angiosarcoma, the rarest subtype of angiosarcoma, exhibits epithelial-like morphology on histopathology, making it difficult to distinguish from other epithelial tumors such as adenocarcinoma or mesothelioma. Due to its rarity, data on epithelioid angiosarcoma are limited to sporadic case reports. In our case, the histological and immunohistochemical profiles of the cardiac and jejunal tumors were similar, raising the possibility that one represented metastasis of the other. However, no established molecular modalities exist to definitively distinguish between primary and metastatic angiosarcoma, and thus, the possibility of synchronous primary tumors cannot be excluded.

To our knowledge, there are few reports of cases in which cardiac angiosarcoma and a visceral lesion, such as a small bowel tumor, are identified concurrently at the time of diagnosis. For example, in Poonia et al.’s review [2], while metastases were present in 89% of left atrial angiosarcoma cases at diagnosis, the literature does not clearly document cases in which gastrointestinal lesions were found simultaneously as separate tumor sites, underscoring the rarity of our case.

Furthermore, compared with previously reported cases of left atrial angiosarcoma, our case is notable for its initial presentation with recurrent cerebral infarctions and a relatively small cardiac tumor. Stergioula et al. [3] reported a median tumor size of approximately 6 cm in a review of primary cardiac angiosarcoma cases, and Kanuri and Vegi [4] described typical tumor sizes ranging from 5.9 to 7.2 cm (range: 1.5-17 cm). By contrast, our cardiac tumor measured only about 15 mm, suggesting that recurrent strokes may have enabled earlier detection.

The cardiac tumor was promptly resected following diagnosis, and small bowel resection and postoperative chemotherapy were performed. However, disease progression with bone metastases was observed, and the patient died eight months after surgery. Despite early intervention, the short survival period highlights the aggressive nature of epithelioid angiosarcoma and the limitations of current treatment modalities.

There is currently no standardized treatment for cardiac angiosarcoma. Surgical resection is typically followed by postoperative chemotherapy and radiotherapy; however, survival outcomes remain poor. Reported five-year survival rates range from 11% to 14%, with a median survival time of only five months in patients with metastasis [5,6]. Cardiac angiosarcomas are often diagnosed at an advanced stage, highlighting the importance of early diagnosis and intervention [7]. Further accumulation of clinical data is necessary to improve the prognosis of patients with this rare and aggressive tumor.

## Conclusions

We report a rare case of left atrial epithelioid angiosarcoma presenting with recurrent strokes and concurrent small bowel involvement. The cardiac tumor was resected promptly, but despite additional multimodal therapy, the disease progressed rapidly, and the patient died within eight months. This case illustrates the highly aggressive nature and atypical clinical course of epithelioid angiosarcoma, which presented with embolic events rather than typical cardiac manifestations such as cardiac tamponade or dyspnea. In addition, a minimally invasive approach may offer potential benefits in patients with a limited prognosis by minimizing surgical trauma.
